# Utilities for High-Throughput Analysis of B-Cell Clonal Lineages

**DOI:** 10.1155/2015/323506

**Published:** 2015-10-07

**Authors:** William D. Lees, Adrian J. Shepherd

**Affiliations:** Institute of Structural and Molecular Biology, Birkbeck College, University of London, Malet Street, London WC1E 7HX, UK

## Abstract

There are at present few tools available to assist with the determination and analysis of B-cell lineage trees from next-generation sequencing data. Here we present two utilities that support automated large-scale analysis and the creation of publication-quality results. The tools are available on the web and are also available for download so that they can be integrated into an automated pipeline. Critically, and in contrast to previously published tools, these utilities can be used with any suitable phylogenetic inference method and with any antibody germline library and hence are species-independent.

## 1. Introduction

Today it is possible to perform high-throughput sequencing of antibody repertoires at a depth that enables the molecular response to a pathogen to be examined [1, 2]. A key focus is on the identification of clonal lineages of B-cells undergoing the process of somatic hypermutation in germinal centres and the maturation pathways by which these lineages develop over time. It is anticipated that a greater understanding of development pathways will facilitate effective vaccine design for challenging targets such as HIV [3], as well as supporting research into autoimmune disease [4] and immune reactions to therapeutic agents.

B-cell receptor variable regions, which contain the hypervariable complementary-determining regions (CDRs), are encoded by cellular DNA which is transformed in the developing cell through a process of somatic recombination known as junction rearrangement. In the light chain, this process involves the rearrangement of two gene segments, V and J, while, in the heavy chain, three segments, V, D, and J, are rearranged [5, 6]. One source of antibody diversity arises from the selection of V(D)J gene segments from the germline, which contains multiple segments and alleles at different genetic loci, while further diversity arises from the rearrangement process itself, in which gene segments are truncated and additional nucleotides inserted. In a process usually requiring T-cell activation, naive B-cells having affinity to an encountered antigen proliferate and are subjected to somatic hypermutation, in which additional mutations are introduced into the variable region of descendent cells, and mutated descendants binding with higher affinity to the target antigen are selected [7, 8]. The large number of germline gene segments, and the stochastic nature of the gene rearrangement process, makes it unlikely that two cells will develop identical arrangements: the arrangement locus, or junction, shared by all descendants, therefore acts as a unique fingerprint that can be used to trace clonally related sequences through this process of affinity maturation, although the additional mutations generated by the process of somatic hypermutation introduce uncertainty [9].

A number of tools have been developed to identify the germline gene segments and junction rearrangement underlying a particular sequence. IMGT [10], in particular, is widely used for large-scale analysis of NGS-derived repertoires. While only available as an online service, it is capable of analysing sets of up to 500,000 sequences at a time. It is supported by (and can only be used in conjunction with) a curated set of antibody germline libraries, covering a number of commonly used experimental species. In NGS studies, clonally related families are typically identified from the output of such tools by collecting sequences that share descent from the same V and J germline segments and have high junction sequence identity at the nucleotide or amino acid level [11, 12]. D germline ancestry is generally not considered, as the junction D-segment is often < 10 nt and the germline can be difficult to identify categorically.

There are few tools available for the analysis of clonally related lineages, and the majority of studies published to date rely on in-house software. ClonalRelate [13] enables the identification of clonally related families based on junction analysis results from iHMMune-align [14], but the two tools are limited to heavy chain sequences. Vidjil [15] provides an innovative junction analysis that can be used as a prescreening step but does not provide definitive germline attribution. ARPP [16] uses sophisticated phylogenetic techniques to reconstruct a B-cell lineage from a set of clonally related sequences but is restricted to human sequences, employing a germline library that is integrated into the program. IgTree [17] develops lineage trees using a novel algorithm as opposed to traditional phylogenetic methods and is distributed under a restricted licence. Our aim with this toolset is to provide open-source tools that can be combined with any available methods for junction analysis and inference of descent, without constraints in terms of germline usage or species. We foresee them being used both as part of a high-throughput pipeline and in the preparation of accurate and informative figures for publication.

In developing an automated pipeline for large-scale analysis of clonally related lineages, we identified two use cases which were not addressed by available tooling and which were time-consuming (and potentially error-prone) to carry out by hand, even on a small scale. The first is the inference of a germline sequence from a junction analysis, either for rooting a phylogenetic tree or for determining the most likely germline CDR configuration corresponding to an isolated sequence. This requires accurate alignment of the germline V(D)J sequences and appropriate handling of the intervening N- and P-sequences, where, in some cases, one may wish to leave the inference to phylogenetic analysis, while in other cases one may wish to make the best inference possible from available sequence data, possibly taking account of information from a number of related sequences. The second use case concerns the inference of ancestral intermediates from a phylogenetic tree and subsequent reporting. Here a number of packages are available for ancestral reconstruction, for example, in PHYLIP [18], PAML [19], and HyPhy [20]. While these tools provide substantial value in an analysis, their direct use imposes constraints on sequence identifier names which are frequently incompatible with those encountered in real-life examples and do not support the direct generation of phylogenetic trees and other reports which embody standard numbering schemes such as those used by IMGT [21] or by the Protein Data Bank [22] or otherwise embody understanding of CDR locations. Compiling reports on clonal lineages using these tools is therefore likely to require input and output file reformatting, followed by manual cross-referencing and labelling of position identifiers and CDR locations.

Here we present two tools to assist with these use cases. The tools are species-independent in that they can be used with any desired germline library and are available both as online services and as open-source code for integration into a local pipeline. Well-established open-source packages are leveraged for phylogenetic analysis, sequence manipulation, and results presentation. The first tool,* RevertToGermline*, uses a simple technique to infer the ancestral sequence from which a clonally related sequence is derived. The second,* AnnotateTree*, takes a phylogenetic tree rooted on this ancestral sequence and provides annotated trees and alignments showing intermediate sequences and amino acid transitions, based on inferred ancestral states.

## 2. Materials and Methods

### 2.1. Algorithms and Functionality

RevertToGermline takes as input a junction analysis of a variable region sequence in which the V(D)J germline gene segments are identified and in which the sequences are divided into regions associated with gene segments and with the intervening spacer regions. In an IMGT analysis, this information is encapsulated in the “nt sequences” section of the analysis, and the tool takes its input in the IMGT format. Use of other junction analysis tools is possible, provided that the output is converted to the simple comma- or tab-separated formats used by IMGT: examples are provided in Supplementary Information available online at http://dx.doi.org/10.1155/2015/323506. The output of RevertToGermline is a sequence in which the V(D)J segments are reverted to germline, while the spacer regions are preserved. A germline library (again in FASTA format, with sequence identifiers in IMGT format) is used to obtain germline segments.

Although they are uncommon, in-frame insertions and deletions can arise in the V-region, when compared to germline. The target V-region is therefore aligned against the germline V-gene at the amino acid level. If the target contains an insertion, the inserted codon is inserted into the derived germline at the same point. If the target contains a deletion, the equivalent codon is deleted from the derived germline. This corresponds to a hypothesis that such insertions and deletions most likely have occurred at the time of junction rearrangement and should therefore be included in the derived germline.

**Figure 1:**
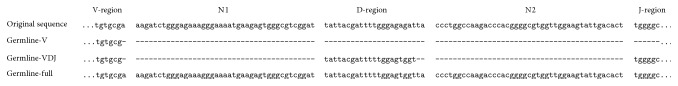
RevertToGermline analysis options. The junction decomposition of a representative heavy chain sequence is shown alongside the three inference options available from RevertToGermline. In the first (V), whole codons in the V-region are reverted to the inferred germline, and other regions are gapped out. In the second (VDJ), whole codons in the D- and J-regions are also reverted to their inferred germlines. In the third (full) option, remaining nucleotides are carried into the inferred germline from the original sequence.

RevertToGermline provides three analysis options, allowing for use in a variety of circumstances (Figure 1). In the first, the germline V-gene is mapped against the input sequence, and remaining nucleotides are gapped. The germline V-gene is trimmed to occupy just that region of the sequence that, according to the junction analysis, is derived from the V-gene in the input sequence. This option provides a convenient root for a phylogenetic tree but yields little information on the likely junction residues of the germline B-cell. The second option maps germline V(D)J sequences in the same manner, putting gaps in just those locations that junction analysis has determined are filled by intervening N and P nucleotides, while the third carries through the N and P nucleotides as well, meaning that there are no gaps in the output sequence. A final option directs RevertToGermline to construct a consensus germline, from germlines inferred for a set of input sequences. Here, in addition to any gaps implied by the above analysis, positions will be gapped if the consensus value is observed in less than 70% of output sequences.

RevertToGermline emits nucleotide sequences that cover whole codons and are aligned on a codon boundary, removing stray nucleotides at the 5′ and 3′ ends. A nucleotide gap in any codon position will be extended to cover the entire codon. These steps allow the output to be consumed without further processing by AnnotateTree and other protein-oriented tools. RevertToGermline can conveniently be run against all sequences in a clonal family. Substantial deviations from consensus, as indicated by gaps in the consensus sequence, may indicate a need to apply stricter criteria when identifying the clonal family members, or postrecombinatorial revision [23]. However in our experience the inferred germlines based on V(D)J sequences provide a useful first approximation to the germline, allowing rapid analysis of multiple clonal families and giving a first indication of changes from germline based on the output from an automated pipeline. In many cases, the germline will not be correctly inferable directly from available sequences, but once the universal common ancestor (UCA) of all sequences is available from ancestral reconstruction, anomalies between the UCA and the inferred germline can be investigated.

Current germline library coverage of allelic variants is known to be incomplete [24]. To assist with the identification of variant alleles unrecorded in the germline library, RevertToGermline will optionally report on the presence of “mutated” positions, insertions, and deletions that are observed in all sequences sharing an imputed V germline ancestor, the implication being that these sequences may have descended from a different V germline not present in the germline library. The analysis is conducted for each germline for which a threshold number of sequences are present in the sample: the threshold is user-configurable in the command-line script and set to 20 in the online service.

Having established an initial germline with RevertToGermline, a rooted phylogenetic tree can be inferred using one of the many established packages such as PHYLIP [18] or IQ-TREE [25]. AnnotateTree uses the resulting tree, and the set of clonally related sequences, to perform the following analyses.

#### 2.1.1. Ancestry Reconstruction

**Figure 2:**
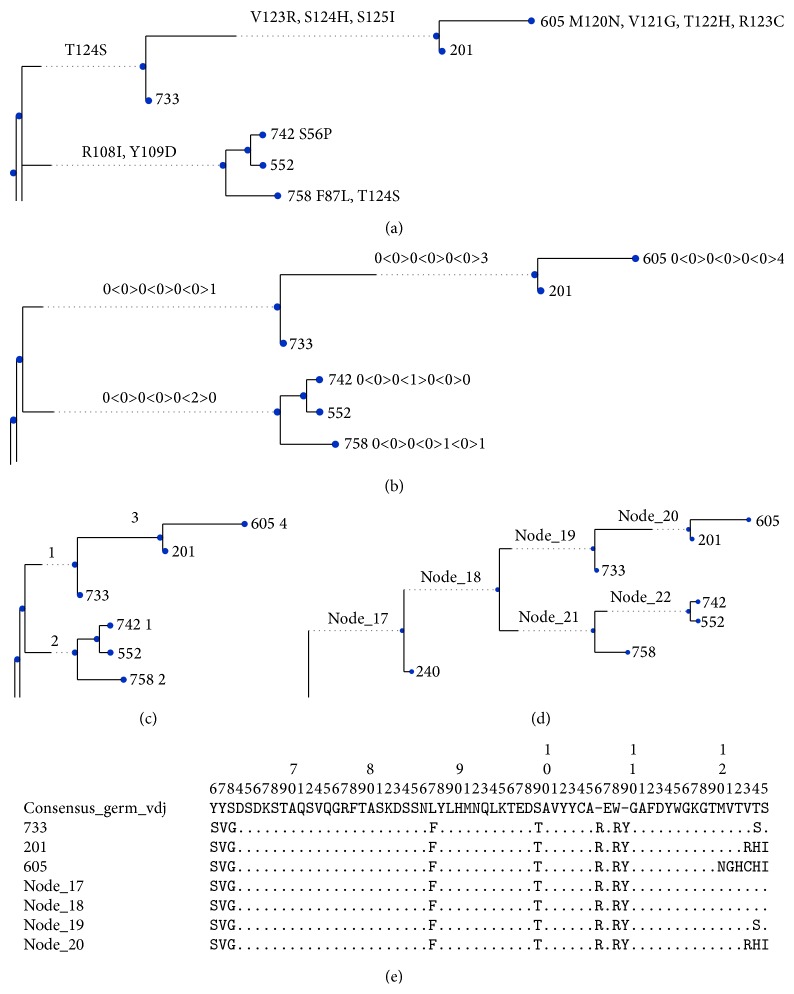
Sample output from AnnotateTree. The samples are taken from the zebrafish repertoire described in more detail in Results and Discussion. (a) Annotation with AA substitutions, using IMGT standard numbering. (b) Summary annotation showing a count of the number of AA substitutions in each FR and CDR using the convention FR1<CDR1>FR2<CDR2>FR3<CDR3>FR4. (c) Annotation showing the total number of AA substitutions on each branch. (d) Tree showing the location of each inferred ancestral node, for cross-reference against AA and nt alignments. (e) Extract from the AA alignment, showing selected nodes from the above trees.

Ancestral sequences are inferred by a maximum likelihood method, using PHYLIP's dnaml [18]. AnnotateTree manages the creation of input files for dnaml and presents the results in a convenient form for the user. As dnaml restricts the format and length of sequence names, the names used in the user's input files are mapped to names acceptable to dnaml during processing and mapped back to the user-provided values in output results. The full dnaml report is available for review. An annotated tree, showing the position of each inferred sequence, is produced, together with an amino acid alignment of submitted and inferred sequences. Further trees are produced showing the total number of amino acid changes along each branch and showing the position of inferred intermediate nodes. Output trees are provided as rendered graphics, and also in Newick format (Figure 2). Nucleotide and amino acid sequences are provided in FASTA format for further analysis. dnaml default settings are used, but in the downloadable software the input parameters are exposed in the file dnaml.ctl and can be changed if required.

#### 2.1.2. Tree Annotation

Amino acid substitutions, determined from ancestry reconstruction, are added to the input tree as node labels. The resulting tree is provided as a rendered graphic (in SVG and PNG formats), and also in Newick format, in which annotations are present as node names.

#### 2.1.3. Position Numbering

The position identifiers of amino acids (as used in the alignment and in the labelling of substitutions) can be flexibly defined by the user. The scheme supports both the PDB-style scheme (e.g., 99, 99A, 99B, and 100) and the scheme used by IMGT [21] (e.g., 111, 111.1, 112.1, and 112), in which it will be noted that insertions can precede their ordinal. Deletions are supported in both schemes. To define the scheme for an alignment, the user specifies the following:

- The position identifier of the first residue in the sequence.
- The position identifiers of any deletions.
- The position identifiers of any insertions occurring* before* the ordinal position identifier (112.1 in the above examples).
- The position identifiers of any insertions occurring* after* the ordinal position identifier (99A, 99B, and 111.1).

#### 2.1.4. CDR Analysis

If, additionally, the locations of the CDRs are specified, each amino acid location within the CDRs is categorised as follows:

- Conserved to germline: the same residue is present at that location in all sequences, including the germline (the first sequence in the submitted sequence file is taken to be the germline).
- Common to trunk: the same residue is present at that location in all sequences except the germline.
- Variation across samples: differing residues are present at that location across the clonal family.

A further tree is also produced, showing a count of the number of amino acid changes in each CDR and framing region, along each branch of the tree.

### 2.2. Case Studies

Three case studies, drawing on previously published sequence sets, are used to demonstrate results from the tools. The sequence sets and full results are provided as Supplementary Information.

### 2.3. PW99 Sequence Set

The PW99 dataset is provided as part of the source distribution of ClonalRelate [13]. Sequences with indeterminate nucleotides were removed. 5′ sequence start positions were aligned by hand and the 3′ end was trimmed to eliminate gaps. This provided 84 distinct sequences with length 375 nucleotides for further analysis. The sequences were analysed by IMGT to determine CDR positions, and the presence of a uniform junction rearrangement was confirmed by review of the IMGT junction analysis. The V-, D-, and J-segments of all sequences were reverted to germline using RevertToGermline, and the consensus of these sequences was used as the root. The phylogenetic tree was inferred by IQ-TREE v1.2.2 using the K3Pu + G4 substitution model, which was determined to be optimal by the software. AnnotateTree was used to derive the annotated tree and ancestral sequences.

### 2.4. Zebrafish Repertoire Sequence Set

An NGS repertoire derived from a sampled zebrafish at each of 5 timepoints was downloaded from https://sites.google.com/site/zebrafishdev/files [26] (in each case the sampled fish labelled “A” was chosen). The sequence set consisted of 22,798 annotated heavy chain reads of 224 nt length, spanning the V-D-J junction. The sequences were analysed by IMGT High V-Quest using the IMGT zebrafish germline library. Clonally related families of productive sequences were determined by clustering the junction nucleotide sequences using CD-HIT [27] with parameters that required identical length and >80% sequence identity. This yielded a total of 381 clusters with two or more members, of which the largest had 127 distinct junction sequences. The full database of 22,798 reads was queried for junction sequences from that cluster that occurred at the 2-week timepoint. 84 matching reads were extracted, deduplicated, and trimmed at the 5′ and 3′ ends using HyPhy [20], yielding 59 distinct sequences of length 208 nt. The IMGT junction analysis of this set was reviewed, and 15 sequences which did not match the consensus V-gene IGHV9-2^*∗*^01 or the consensus J-gene IGHJ2-1^*∗*^0 were removed, as was one further unproductive sequence. The V-, D-, and J-segments of remaining sequences were reverted to germline using RevertToGermline, and the consensus of these reversions was used as the root. The phylogenetic tree was inferred by IQ-TREE v1.2.2 using the K2P + G4 substitution model, which was determined to be optimal by the software. AnnotateTree was used to derive the annotated tree and ancestral sequences.

### 2.5. HIV-Neutralizing Antibodies Sequence Set

The heavy chain sequence set and inferred tree were downloaded in Nexus format from the Supplementary Information of the original study [28] and converted to separate FASTA and Newick format files using HyPhy [20] before processing with AnnotateTree. As insertions are developed in the course of the lineage and gaps are not properly handled by PHYLIP dnaml, the UCA inferred by dnaml was compared against the nearest sequence in the phylogenetic tree (038-234314) and matching gaps were created to replace ancestral nucleotides incorrectly inferred by dnaml.

## 3. Results and Discussion

### 3.1. VH4-34 Lineage in the Human Tonsil

**Figure 3:**
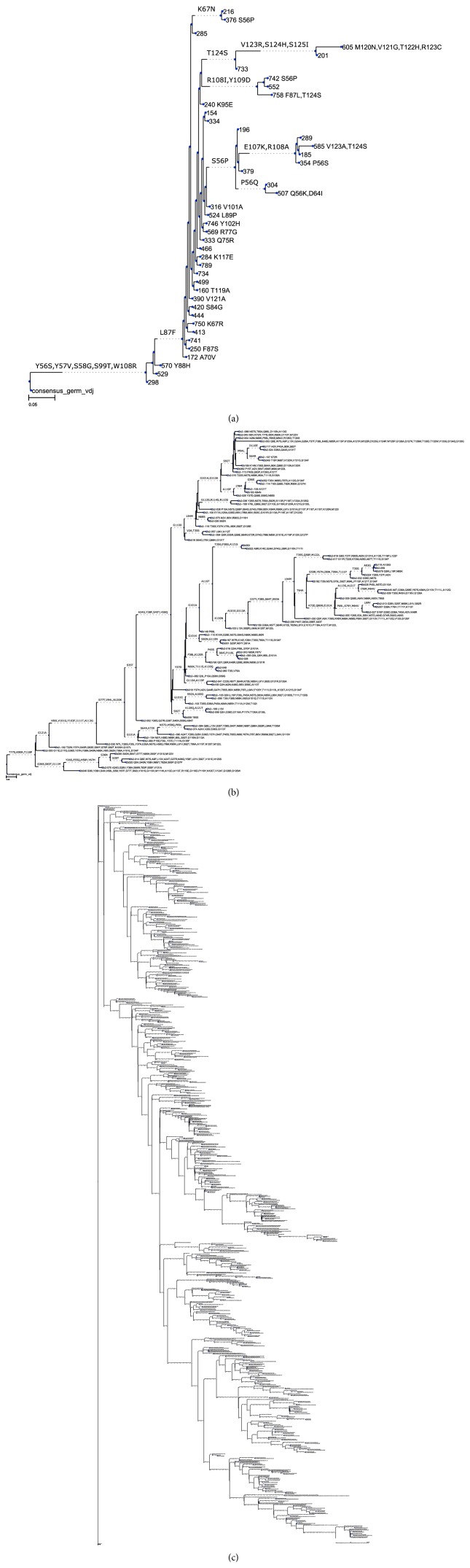
Phylogenetic trees for case studies discussed in this paper. (a) VH4-34 lineage in the human tonsil (84 sequences); (b) heavy chain clonal family from a 2-week-old zebrafish (43 sequences); (c) developmental lineage of the HIV bnAb CAP256-VRC26 over 8 timepoints (692 sequences). Larger copies of these trees, plus other output from AnnotateTree, can be found in the Supplementary Information.

A characterisation of VH4-34-encoded antibodies isolated from tonsils of healthy human subjects has been previously described [29], and the PW99 dataset, consisting of 99 sequences isolated from a single sample and known to be derived from the same V-D-J rearrangement, has been used in a previous analysis of clonal diversity [13]. A phylogenetic tree inferred by IQ-TREE and annotated by AnnotateTree (Figure 3 and Supplementary Information) shows broad development from the germline, with the absence of CDR-based mutations in the trunk and relatively short development pathways suggesting a repertoire formed by primary rearrangement.

### 3.2. B-Cell Heavy Chain Development in the Juvenile Zebrafish

In a contrasting study, we isolated and analysed a clonal lineage of 43 partial V-gene sequences isolated from a 2-week-old zebrafish [26]. The pattern is again one of broad development (Figure 3 and Supplementary Information) but the presence of conserved mutations in the trunk is suggestive of more focussed development. The development of substitutions in the framing regions, particularly FR4, compared to the CDRs, is notable.

### 3.3. Developmental Pathway of HIV-Neutralizing Antibodies

The lineage of neutralizing antibody CAP256-VRC26, which binds to the variable regions 1 and 2 of the HIV-1 envelope, has been described [28]. We analysed the heavy chain lineage sequence set, consisting of 692 sequences, using the inferred tree provided by the authors, which is rooted on the IGHV3-30^*∗*^18 germline (Figure 3 and Supplementary Information). This sequence set contains samples from 8 timepoints over a 4-year period. A number of insertions can be seen in the amino acid alignment, and it will be noted that insertions are not annotated on the output tree. This is because dnaml treats sequence gaps as unknown nucleotides and therefore does not represent them in intermediate sequences [30]. The UCA inferred by dnaml was corrected to restore the gaps observed in the phylogenetically closest sequence (see Section 2.5). V, D, and J segments were then reverted to germline using RevertToGermline, and the resulting output was observed to be in entire agreement with the UCA, indicating that V, D, and J segments in the UCA represent original germline values and showing that the lineage can be traced back close to the germline, although prior unobserved changes in the N-regions cannot be ruled out.

### 3.4. Availability

Both tools are available online at http://cimm.ismb.lon.ac.uk/pat. The tools are written in Python 2.7 with BioPython [31] and the ETE Toolkit [32]: source code and installation instructions for command-line scripts may be downloaded from the above location.

## 4. Conclusion

The tools described in this paper were developed to meet the needs of our own work but we are making them publicly available as a small contribution towards the important goal of developing an accepted standard for the analysis of antibody repertoires. Although this work is primarily directed at the analysis of B-cell clonal families, AnnotateTree may be useful for the creation of alignments, annotations, and ancestral reconstructions of other sequences.

Given the ever-increasing volumes of data, and the increasingly widespread use of NGS by experts in other fields, we feel that it is important to create tools that can be accessed conveniently online for casual use but also installed locally as part of a high-throughput automated pipeline, avoiding the need to work around the volume limitations or queue sizes of online shared services. Given the rapid development of knowledge in the field, toolsets should be as open as possible so that germline libraries and third-party components can be readily updated. Our utilities embody these principles.

## Supplementary Material

The Supplementary Information contains input files and output files from TreeAnnotate for the three case studies described in the article.
