# Supplementary material for: Utilities for High-Throughput Analysis of B-Cell Clonal Lineages
Source: J Immunol Res. 2015 Oct 7;2015:323506. doi: 10.1155/2015/323506 (PMC4617424; doi:10.1155/2015/323506)
Supplement: Supplementary file 1 — The Supplementary Information contains input files and output files from TreeAnnotate for the three case studies described in the article. [file 323506.f1.pdf]

Utilities For High-Throughput Analysis of B-Cell Clonal Lineages
Supplementary Information

Graphical Output from Case Studies

VH4-34 lineage in the human tonsil

Amino Acid Substitutions

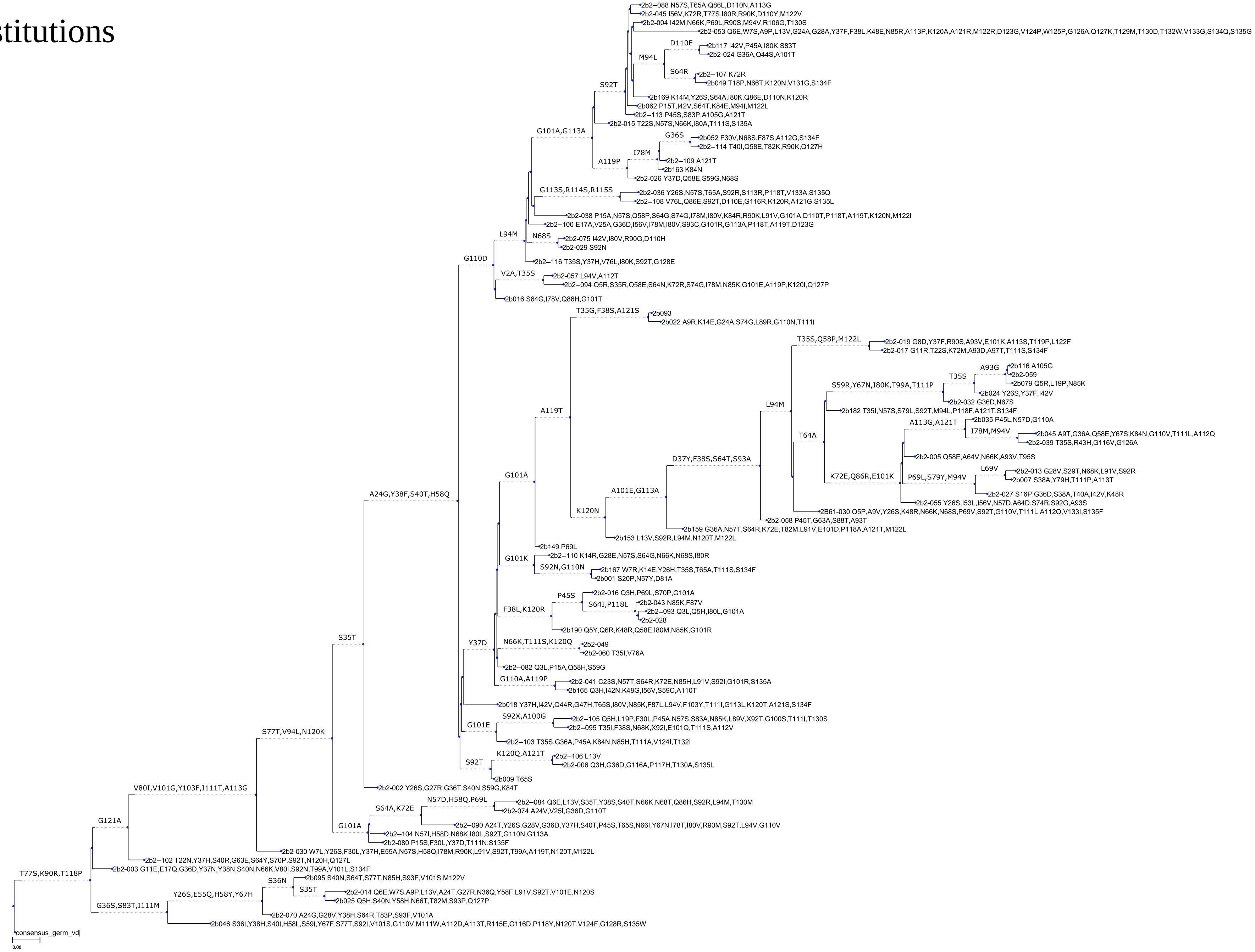

Substitutions by Region

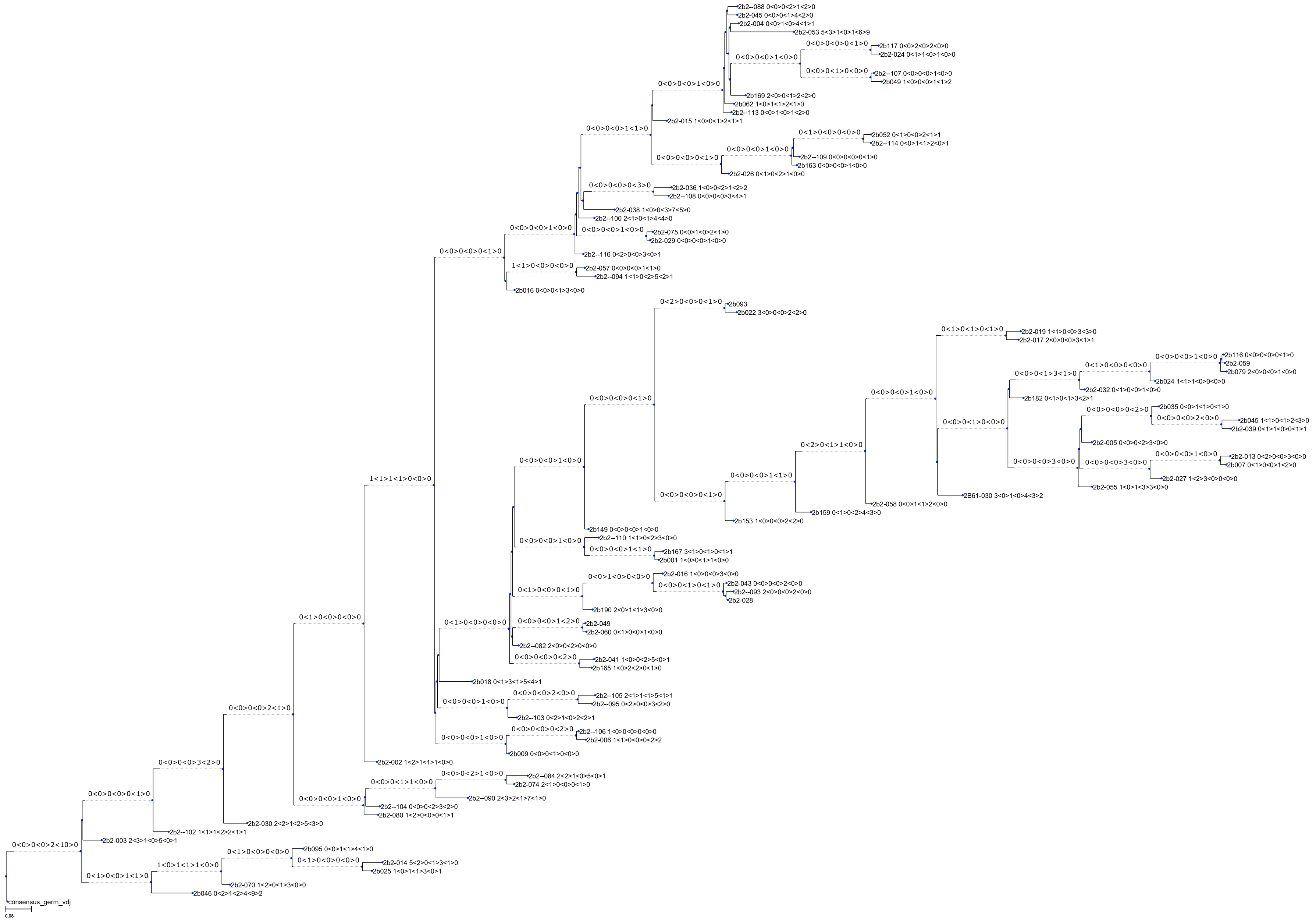

Total Amino Acid Substitutions

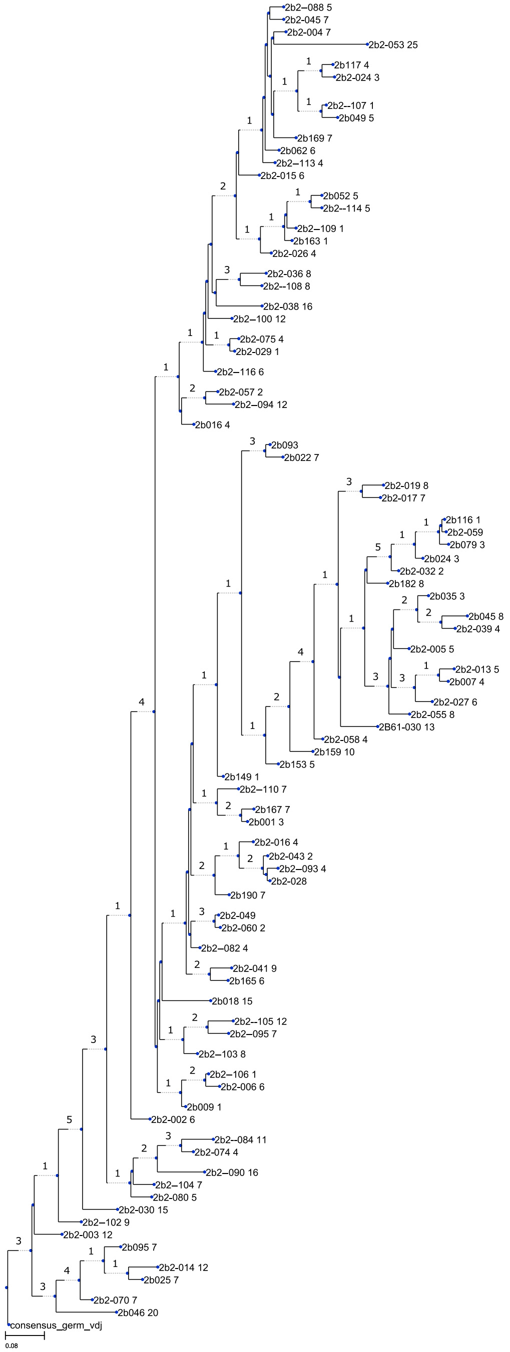

Intermediate Node Names

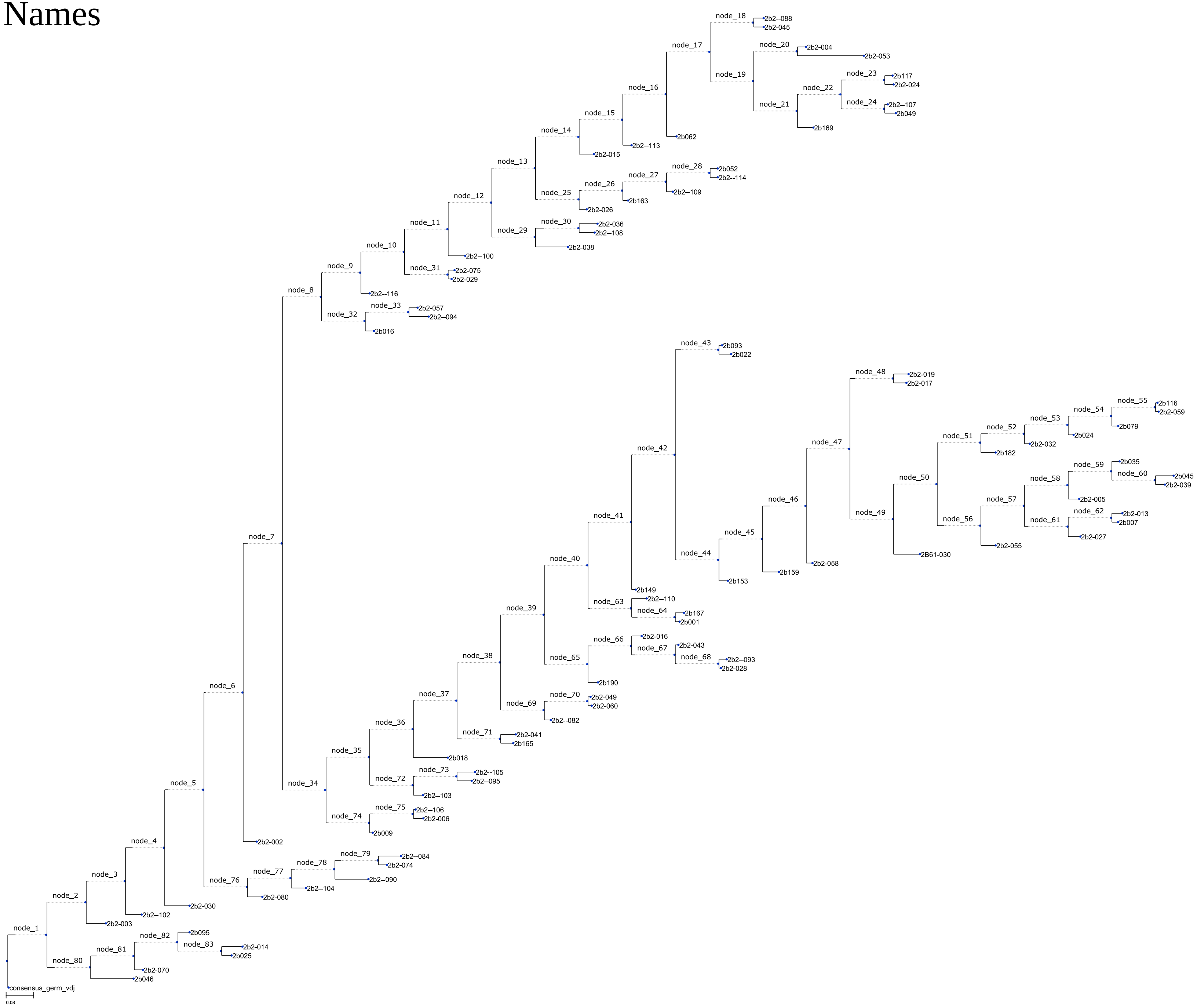

CDR Change Analysis

	Conserved to Germline	Common to All	Variations Across Samples
CDR1			27:GR,28:AEGV,29:ST,30:FLV,35:GIRST,36:ADGINQST,37:DFHNY,38:AFHLNSY
CDR2			56:IV,57:DINSTY,58:DEFHLPQY,59:CGIRS,63:AEG,64:ADGINRSTVY,65:AST
CDR3		107G,108L,109S	105:AG,106:GR,110:ADEGHNTVY,111:AILMNPSTW,112:ADGQTV,113:AGLPRST,114:RS,115:ERS,116:ADGRV,117:HP,118:AFLPTY,119:APT,120:AHIKNQRST,121:AGRST,122:FILMRV,123:DG,124:FIPV

Heavy chain clonal family from a 2-week old zebrafish

Amino Acid Substitutions

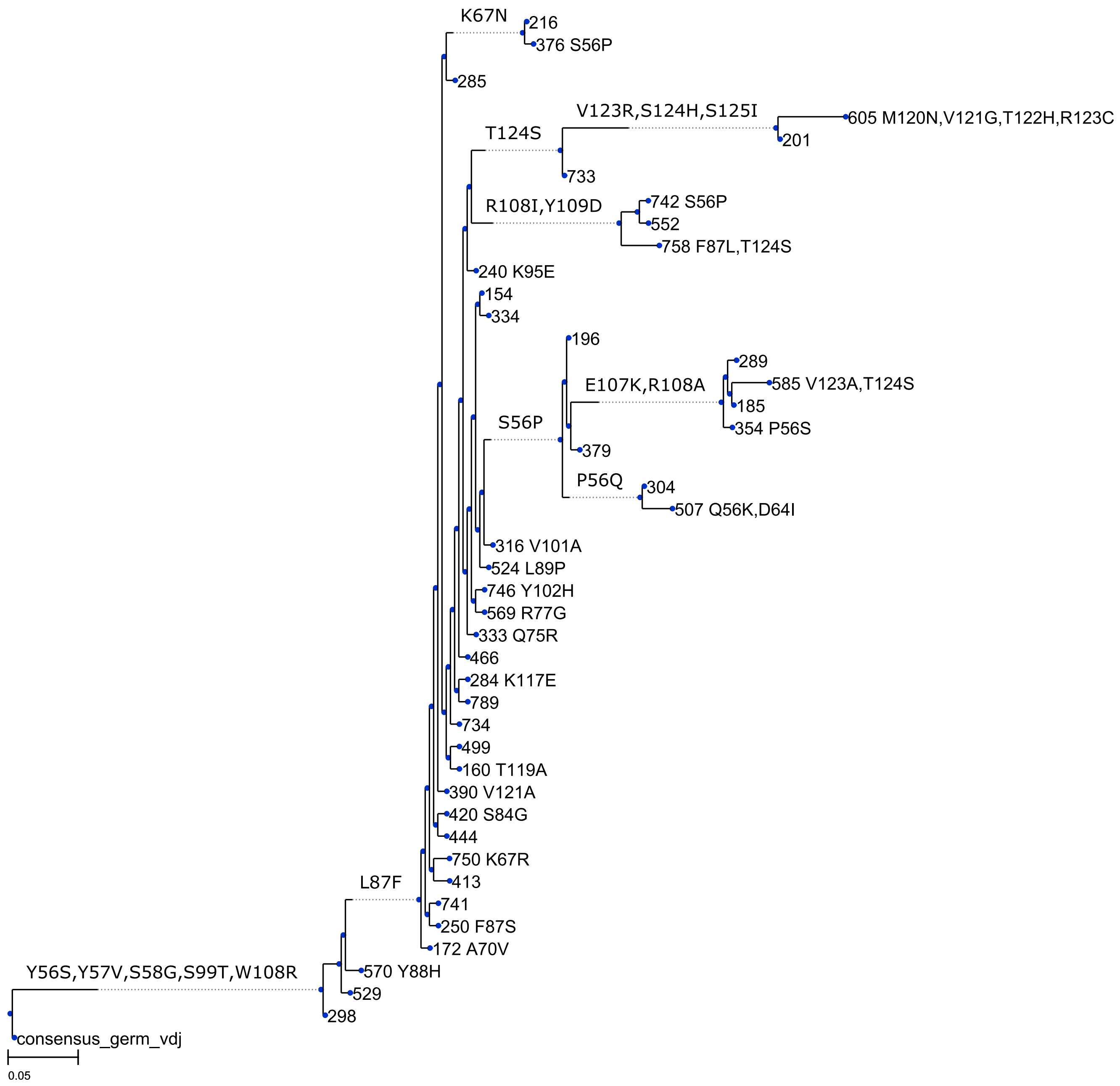

Substitutions by Region

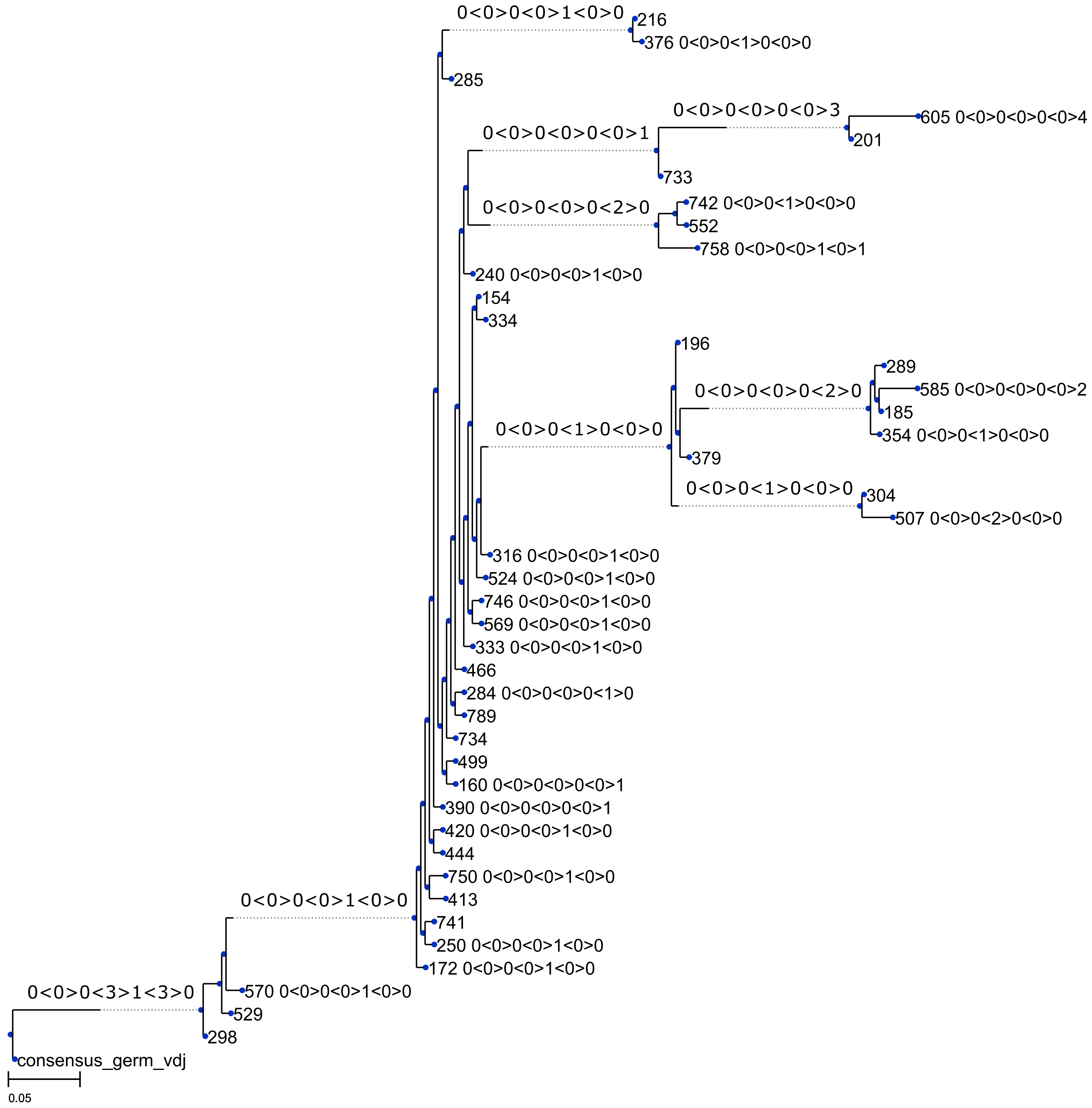

Total Amino Acid Substitutions

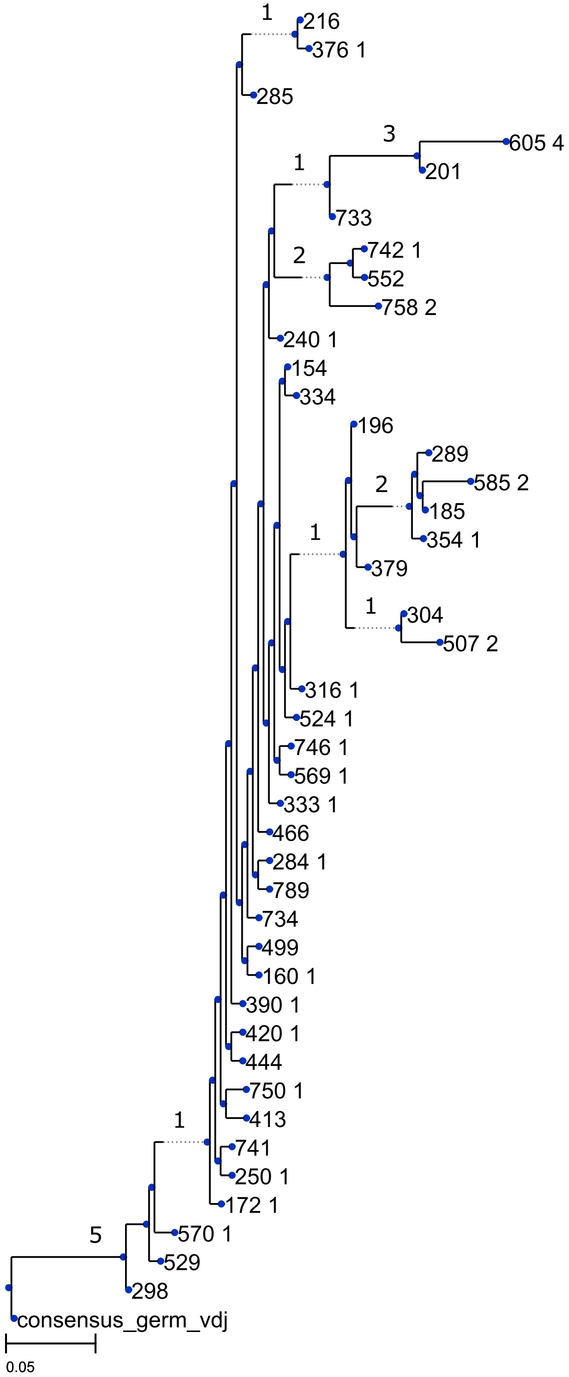

Intermediate Node Names

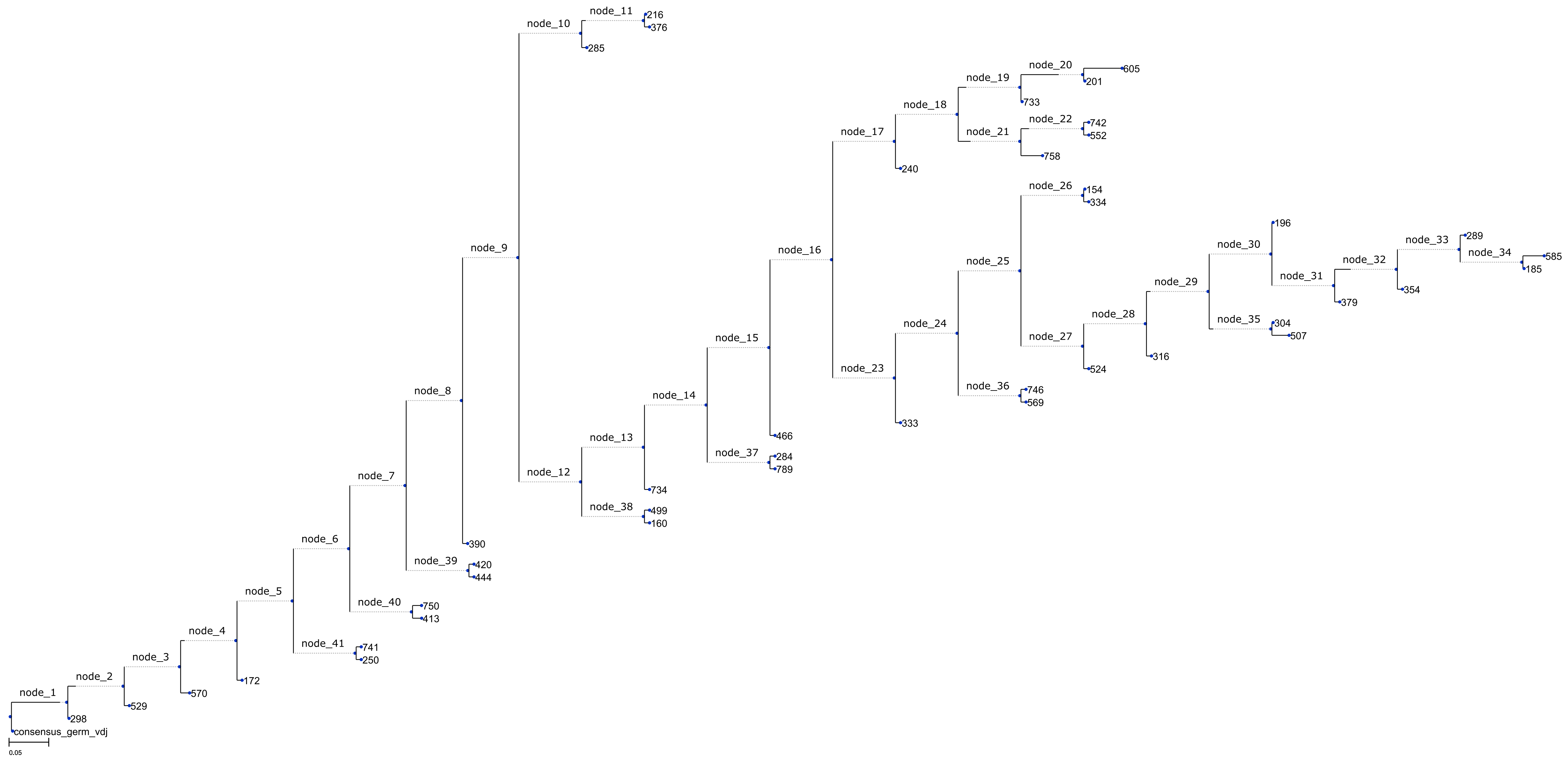

CDR Change Analysis

	Conserved to Germline	Common to All	Variations Across Samples
CDR1			
CDR2	65S	57V,58G	56:KPQS,64:DI
CDR3	105A,110G,111A,112F,113D,114Y,115W,116G	106R	107:EK,108:AIR,109:DY,117:EK

Developmental lineage of the HIV bnAb CAP256-VRC26 over 8 timepoints

Amino Acid Substitutions

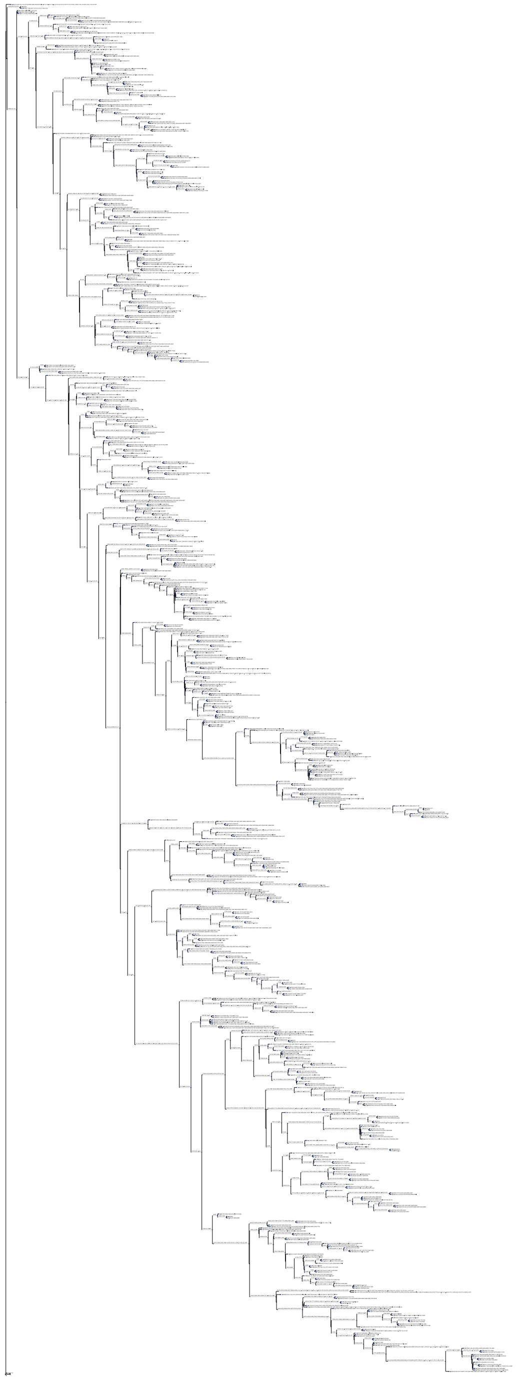

Substitutions by Region

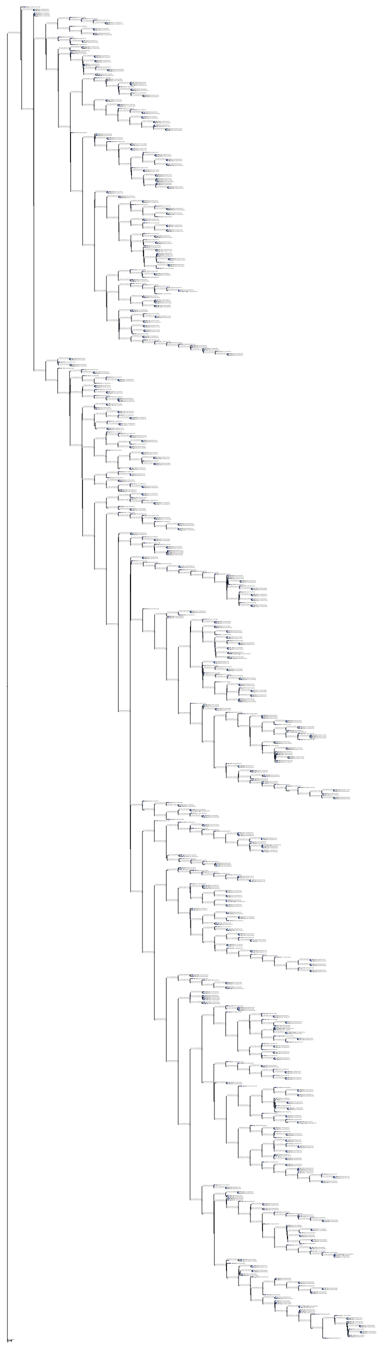

Total Amino Acid Substitutions

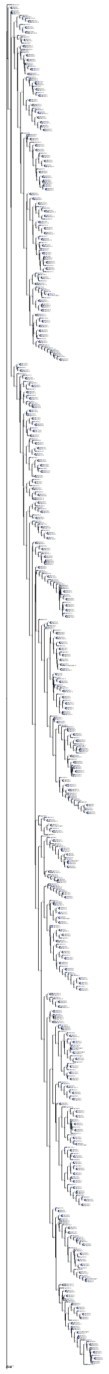

Intermediate Node Names

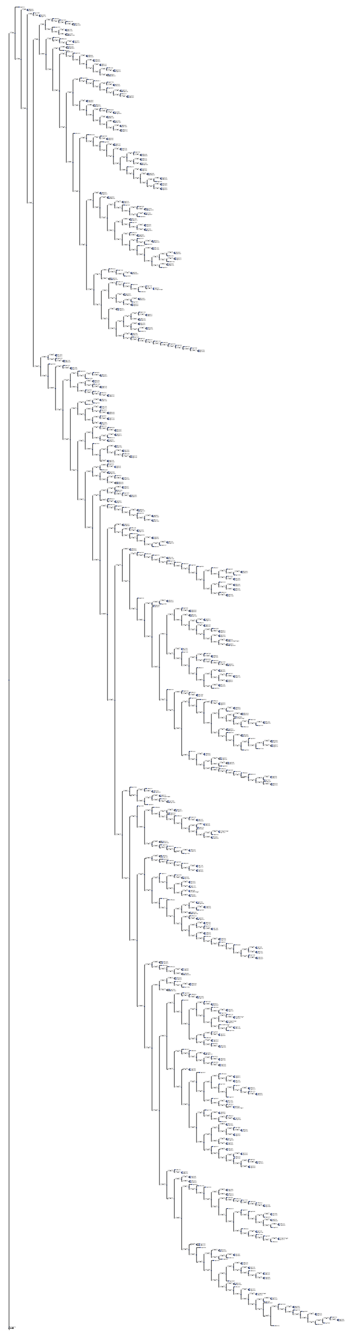

CDR Change Analysis

	Conserved to Germline	Common to All	Variations Across Samples
CDR1			27:-ADEGHKQRVXY,28:-FILV,29:-AEGIKMNPRSTY,30:-FILSVY,35:ACDEGIKNRSTVXY,36:ADEFGHIKNPQRSTVXY,37:ACFHNSTVY,38:ACDEFGHIPSTVWXY
CDR2			56:-EILMTVX,57:-AGHLNPRSTWX,58:-DFGHKLNPSTXY,59:-ADEGHNPRSV,62:-ADEGRS,63:-ADGHIKMNQRSTVXY,64:-ADEFGHIKLNQRSTXY,65:*-DEFIKMNQRSTXY
CDR3		112.11Y	105:ATV,106:KR,107:DE,108:AEHILMPQRSVX,109:ADGHQRX,110:ADEFGHIKLQVY,111:ADEFGHIKLMNRSTVWY,111.1:ADEKQS,111.2:CDEIKNPRSTV,111.3:-EIMVX,111.4:-D,111.5:DEGLM,111.6:AELQRTVY,111.7:RW,111.8:-AEGKPQRSTW,111.9:ALST,111.10:-P,111.11:DEHNTY,111.12:-NXY,112.10:CEY,112.9:-ADEGNSV,112.8:-FILMW,112.7:-W,112.6:-AGRSWY,112.5:-DEIKNRSTVY,112.4:-ADEGHKNPQRSVWX,112.3:-DN,112.2:ACDFGHILQRSVY,112.1:LPT,112:ACGILVY,113:AEHIKLPQRTV,114:ADEGHKNQSTV,115:AFPQRSTVWY,116:GHKPQRT,117:-GT
